# Micro-RNA-338-3p Promotes the Development of Atherosclerosis by Targeting Desmin and Promoting Proliferation

**DOI:** 10.1007/s12033-021-00341-8

**Published:** 2021-06-07

**Authors:** Shiran Yan, Jing Chen, Teng Zhang, Jian Zhou, Ge Wang, Yanfen Li

**Affiliations:** 1 Department of Cardiology, Heze Municipal Hospital, No. 2888, Caozhou West Road, Heze, 274000 China; 2 Department of Internal Medicine, Licun Township Health Center, Heze, 274038 China; 3 Gaozhuang Town Central Health Center, Heze, 274000 China; 4 Department of Central Laboratory, Affiliated Beijing Chaoyang Hospital of Capital Medical University, Beijing, 100043 China

**Keywords:** Atherosclerosis, miR-338-3p, Vsmcs, Phenotype

## Abstract

Atherosclerosis (AS) is a dynamic and multi-stage process that involves various cells types, such as vascular smooth muscle cells (VSMCs) and molecules such as microRNAs. In this study, we investigated how miR-338-3p works in the process of AS. To determine how miR-338-3p was expressed in AS, an AS rat model was established and primary rat VSMCs were cultured. Real-time polymerase chain reaction was performed to detect miR-338-3p expression. Markers of different VSMC phenotypes were tested by Western blot. Immunofluorescent staining was employed to observe the morphologic changes of VSMCs transfected with miR-338-3p mimics. A dual luciferase reporter assay system was used to verify that desmin was a target of miR-338-3p. To further identify the role of miR-338-3p in the development of AS, VSMC proliferation and migration were evaluated by EdU incorporation assay, MTT assay, and wound healing assay. miR-338-3p expression was upregulated in the aortic tissues of an AS rat model and in primary rat VSMCs from a later passage. The transfection of miR-338-3p mimics in VSMCs promoted the synthetic cell phenotype. Bioinformatics analysis proposed desmin as a candidate target for miR-338-3p and the dual luciferase reporter assay confirmed in vivo that desmin was a direct target of miR-338-3p. The MTT and EdU incorporation assay revealed increased cell viability when miR-338-3p mimics were transfected. The increased expression of PCNA was a consistent observation, although a positive result was not obtained with respect to VSMC mobility. In AS, miR-338-3p expression was elevated. Elevated miR-338-3p inhibited the expression of desmin, thus promoting the contractile-to-synthetic VSMC phenotypic transition. In addition to morphologic changes, miR-338-3p enhanced the proliferative but not mobile ability of VSMCs. In summary, miR-338-3p promotes the development of AS.

**Supplementary Information:**

The online version contains supplementary material available at 10.1007/s12033-021-00341-8.

## Introduction

In cardiovascular diseases, atherosclerosis (AS) is the main cause of morbidity and mortality [1]. AS is a dynamic and multi-stage process, and includes dyslipidemia; an inflammatory response; endothelial cell (EC) dysfunction; vascular smooth muscle cell (VSMC) proliferation, differentiation, and migration; and the formation of foam cells, occurring simultaneously or successively. These events result in macroscopic changes – namely, the formation of fatty streaks that evolve into fibrous plaques and atheromatous plaques [2]. The narrowing of blood vessels caused by these aforementioned changes significantly increases the likelihood of a heart attack or stroke [3]. Several well-accepted risk factors such as hypercholesterolemia, hypertension, tobacco smoking, diabetes, obesity, and hereditary factors will initiate or accelerate the pathological process of AS [4].

Various hypotheses have been put forward to try to account for the mechanisms underlying AS, such as the “response-to-injury” theory, although it is still obscure [5]. A variety of cell types participate in the initiation and progression of AS, including inflammatory cells, ECs, and VSMCs. During the development of the human body, VSMCs primarily exist in two different phenotypes: contractile and synthetic [6]. Synthetic VSMCs predominate in the embryonic and neonatal periods with active proliferative capacity; while contractile VSMCs are mainly found in adults and deprived of their ability to proliferate [7–10]. VSMCs are key cellular components of the vessel wall, and normally constitute the muscular structure by a “contractile” phenotype to regulate vascular tone and elasticity [11]. When pathological processes occur, adaptive changes are made, which are accompanied by a phenotype shift into a “synthetic” phenotype, which is characterized by the decreased expression of contractility-associated genes such as SM α-actin (α-SMA), smooth muscle myosin heavy chain (SM-MHC), and desmin [12]. VSMC proliferation is part of the initiation and the progression of AS [13]. When stimulated by a dysfunctional endothelium and inflammatory factors, VSMCs differentiate, proliferate, and migrate into intima [14, 15]. At the fatty streak stage, VSMCs begin to differentiate into foam cells [6].

MicroRNAs (miRNAs) refer to a subset of non-coding RNAs of 18–22 nt. The most widely known function of miRNAs is that it silences the expression of mRNA by pairing with target sequences situated in the 3’ UTR of mRNA [16]. Having penetrated into almost all fields of research, the significance of miRNAs in cardiovascular diseases has drawn increased attention. AS consists of multiple stages, and each involves complex cellular and molecular elements, including a variety of miRNAs. For example, miR-148a, miR-128-1, miR-130b, and miR-301b were found to be important regulators of cholesterol homeostasis [17]. As protective miRNAs, miR-155 and miR-221/222 weaken the adhesion of T cells to activated ECs and they reduce EC migration by targeting the transcription factor Ets-1 and its downstream genes, such as VCAM-1 [18]. In addition, the switch of VSMCs from a “contractile” phenotype into a “synthetic” phenotype, as well as the development of plaques, requires miRNA participation [19].

Of the many molecules involved in cardiovascular physiopathology, miR-338 is emerging as a key player in the molecular network. For example, miR-338-3p is upregulated in left ventricular assistive device (LVAD)-supported versus “control” (failing explanted) hearts [20]. Also, miR-338 has been demonstrated to be elevated in rat hearts under prolonged hypoxia/ischemic injury [21]. In AS, miR-338-3p was found to promote ox-LDL-induced EC cell injury by targeting BAMBI and activating the TGF‐β/Smad pathway [22]. In this study, we explored the potential role of miR-338-3p on VSMCs in the progression of AS.

## Methods

### Cell Culture and Transfections

Human VSMC and 293-T cell lines were purchased from the American Tissue Culture Collection (ATCC, Manassas, VA, USA) and cultured in Dulbecco’s modified Eagle medium (DMEM; Thermo Fisher Scientific, Waltham, MA, USA) supplemented with 10% fetal bovine serum (FBS; Thermo Fisher Scientific), 1% penicillin/streptomycin (Thermo Fisher Scientific). Cells were grown at 37 °C in the presence of 5% CO_2_. The medium was replaced every 24 ~ 48 h. The miR-338-3p mimic, the negative control (NC), and the miR-338-3p inhibitor sequences were purchased from Genechem (Shanghai, China) and transfected into cells using lipofectamine 2000 (Thermo Fisher Scientific).

### Animal Experiment

The ApoE^−/−^ and wild-type rat (Sprague–Dawley strain, 6 weeks old) were obtained from the Shanghai Model Organisms Center. A total of 15 rats (10 ApoE^−/−^ and 5 wild-type) with similar body weights were included in this study. The ApoE^−/−^ rats were randomly divided into two groups: those fed a high-fat diet (HFD 21% fat, 20% protein and 0.15% cholesterol) and those fed a normal diet. After 12 weeks, all animals were sacrificed for further study.

### Primary Rat VSMC Isolation and Culture

Approximately 5 cm segments of thoracic aortas of Sprague–Dawley rats (150 ~ 250 g) were isolated. Afterwards, a single-cell suspension was obtained using an enzymatic dispersion technique, as previously described [7]. The segments were immediately placed in DMEM and washed three times. The fat and connective tissues were stripped and separated from the media. The remaining specimen (mainly media) was then minced and incubated with a trypsin and collagenase II solution at 37 °C to obtain a single-cell suspension. Then, the cells were centrifuged and re-suspended in serum-free DMEM. Twenty-four hours later, the medium was replaced with DMEM containing 10% FBS and 1% penicillin/streptomycin. All experiments were done with cells from passages 3 through 10.

### Western Blot

Total proteins from tissues or cells were extracted by lysing in RIPA lysis buffer containing a protease inhibitor (Applygen, Beijing, China). The proteins were separated via sodium dodecyl sulfate polyacrylamide gel electrophoresis (SDS-PAGE) and then transferred to polyvinylidene fluoride (PVDF) membranes (MilliporeSigma, Burlington, MA, USA). After blocking with Tris-buffered saline containing 0.05% Tween-20 (TBST) and 5% non-fat dry milk or 5% BSA for 1 h at room temperature, the membranes were incubated with anti-α-SMA (1:1000; #19,245; Cell Signaling Technology, Danvers, MA, USA), anti-desmin (1:1000; #5332; Cell Signaling Technology), anti-PCNA (1:500; sc-56; Santa Cruz Biotechnology, Inc., Dallas, TX, USA), and anti-β-actin (1:1000; sc-47778; Santa Cruz Biotechnology, Inc.) antibodies at 4 °C overnight, followed by incubation with corresponding horseradish peroxidase (HRP)-conjugated secondary antibodies for 1 h at 25 °C. β-actin was used as an internal control. Proteins were detected with ECL chemiluminescence and their intensity was analyzed with ImageJ software (National Institutes of Health, Bethesda, MD, USA).

### Immunofluorescent Staining and Confocal Microscopy

The cells were seeded in 24-well plate and grown for 24 h. When they reached 60% ~ 70% confluency, the cells were fixed with 4% paraformaldehyde for 20 min and were permeabilized with 0.3% Triton X-100 for 20 min at 25 °C. Non-specific binding sites were blocked with 1% bovine serum albumin (BSA; Sigma-Aldrich Co., St. Louis, MO, USA) containing 0.3% Triton X-100 in phosphate-buffered saline (PBS) for 1 h. Following incubation with anti-desmin (1:300; sc-23879; Santa Cruz Biotechnology, Inc.) in 0.1% BSA overnight at 4 °C, the cells were incubated with goat anti-mouse antibodies (Alexa Fluor^®^ 488; 1:300; ab150113; Abcam plc, Cambridge, UK) for 1 h at 25 °C. The nuclei were stained with DAPI. Images were obtained with a confocal laser scanning microscope (Leica TCS SP5 MP; Leica Microsystems, Wetzlar, Germany).

### RNA Extraction and Real-Time PCR

The expression of miR-338-3p was measured by quantitative real-time polymerase chain reaction (qRT-PCR). Briefly, the total RNA was extracted using a miRcute miRNA Isolation Kit (Tiangen Biotech, Shanghai, China) according to the manufacturer’s protocol. The expression levels of miRNA were analyzed using a Taqman miRNA assay (Applied Biosystems, Foster City, CA, USA) and normalized to U6 small nuclear RNA expression. For the analysis of desmin, total RNA was extracted with TRIzol reagent (Thermo Fisher Scientific), according to the manufacturer’s instructions. Reverse transcription reactions were incubated in a Veriti 96-well Thermal Cycler (Thermo Fisher Scientific) using 5 × All-In-One MaterMix (Applied Biological Materials, Vancouver, BC, Canada). Real-time PCR was performed in an ABI 7500 real-time PCR system (Thermo Fisher Scientific) using the Maxima SYBR Green qPCR Master Mix (Thermo Fisher Scientific). All PCR reactions were run in triplicate. GAPDH was used as internal control. Relative gene expression was determined using the comparative CT (2^−ΔΔCt^) method.

### Dual Luciferase Reporter Assay System

The potential binding targets of miR-338-3p were predicted using the TargetScan (http://www.targetscan.org/) and miRDB (http://mirdb.org) databases. The sequences of segments with wild-type or mutant seed regions of DES were synthesized and cloned into pmirGLO Vectors (Promega Corporation, Madison, WI, USA). 293-T cells grown in 96-well plates (1 × 10^4^ cells/well) were transfected with different combinations of miR-338-3p (or miR-NC, 50 nM) and pmirGLO (vector, pmirGLO-DES-3’-UTR Wt or pmirGLO-DES-3’-UTR Mut; 50 ng/well) using Lipofectamine 2000 (Thermo Fisher Scientific). Cells were harvested for 24 h post-transfection, and luciferase activities were analyzed using = the dual luciferase reporter assay system (Promega Corporation).

### Cell Proliferation Analysis by MTT Assay

VSMCs were cultured in a 96-well plate with 100 μL of 5 × 10^4^/mL cells for each well. After transfection, cell proliferation was determined at 0 h, 24 h, 48 h, and 72 h, and each treatment was repeated in five wells. Briefly, 20 μL of MTT solution (5 mg/mL) was added to the wells, followed by incubation at 37 °C for 4 h. After removing the reagent, 150 µL of DMSO was added to each well. The plate was shaken gently for 10 min, and the cell viability was represented as the absorbance value measured at 490 nm, as determined by a Biotek Synergy 4 microplate reader (BioTek Instruments, Inc., Winooski, VT, USA).

### EdU Incorporation Assay

For EdU analysis, the nuclear DNA was counterstained using Hoechst 33,342, and EdU-positive images were captured by a fluorescence microscope (80i; Nikon, Inc., Tokyo, Japan).

### Wound-Healing Assay

The wound-healing assay was performed to probe for collective cell migration. Briefly, VSMCs were plated in six-well plates at a density of 4 × 10^5^ cells/well. After adherence, cells were transfected with the indicated expression vectors. After being rinsed with medium to remove the unattached cells, the confluent layer of cells was scratched with a sterile tip to create an artificial wound. Cell migration to the wounded gap was then monitored by microscopy after 24 h and 48 h. The wound area (A) at different time was measured using the Image J software. Wound closure (%) was determined according to: Wound Closure % = [(A_0_– A_t_)/ A_0_] × 100 where the area at time zero (A_0_) and the area at indicated time (A_t_) were used to calculate the wound closure percentage.

### Statistical Analysis

All experimental data were processed and analyzed using SPSS 21.0 (IBM Corporation, Armonk, NY, USA) and GraphPad Prism 8 (GraphPad Software, La Jolla, CA, USA). Data were presented as the mean ± standard deviation. The differences between groups were explored using Student’s t-test. Differences were considered statistically significant when *P* < 0.05.

## Results

### miR-338-3p Expression was Upregulated in an AS Rat Model

**Fig. 1:**
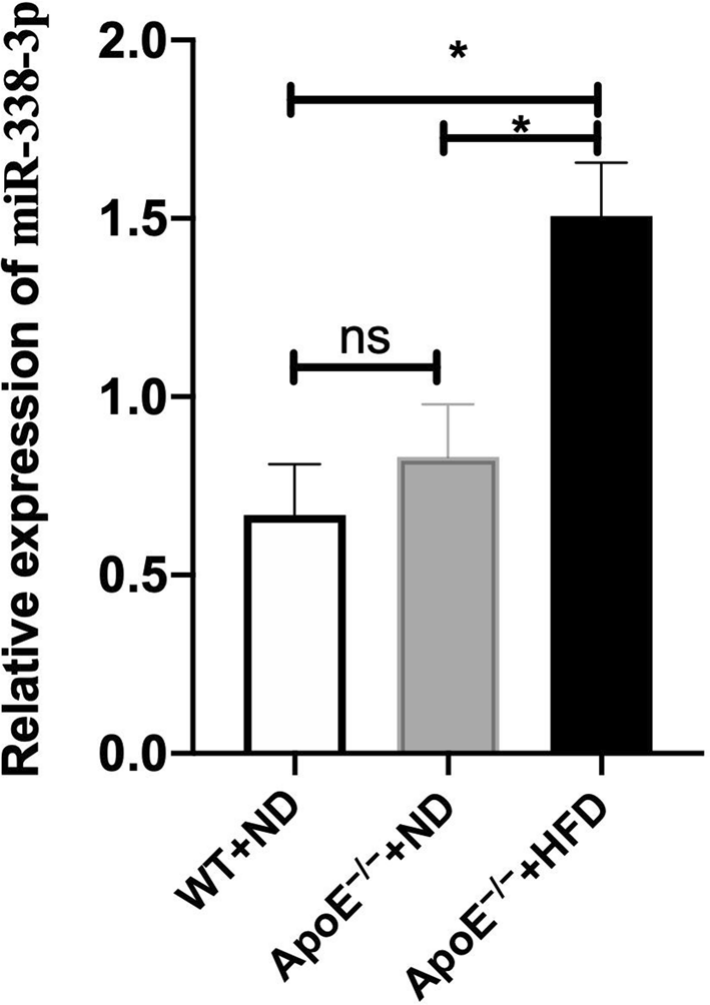
Real-time PCR revealed that miR-338-3p was markedly elevated in aortic tissues obtained from ApoE^−/−^ rats fed a high-fat diet. There was no significant change between rats fed normal diets. ^*^P < 0.05

To identify miR-338-3p expression in AS, we tested the expression level of miR-338-3p using rat aorta tissues. Compared with wild-type and ApoE^−/−^ rats that were fed with a normal diet (ND; Fig. 1), miR-338-3p was markedly elevated in tissues obtained from ApoE^−/−^ rats fed a high-fat diet (HFD), and the relative expression of miR-338-3p was 1.51 ± 0.15 versus 0.67 ± 0.14(wild-type), 1.51 ± 0.15 versus 0. 83 ± 0.15(ApoE^−/−^),respectively.

### VSMCs of Later Passage Showed a Relatively High Level of miR-338-3p

**Fig. 2:**
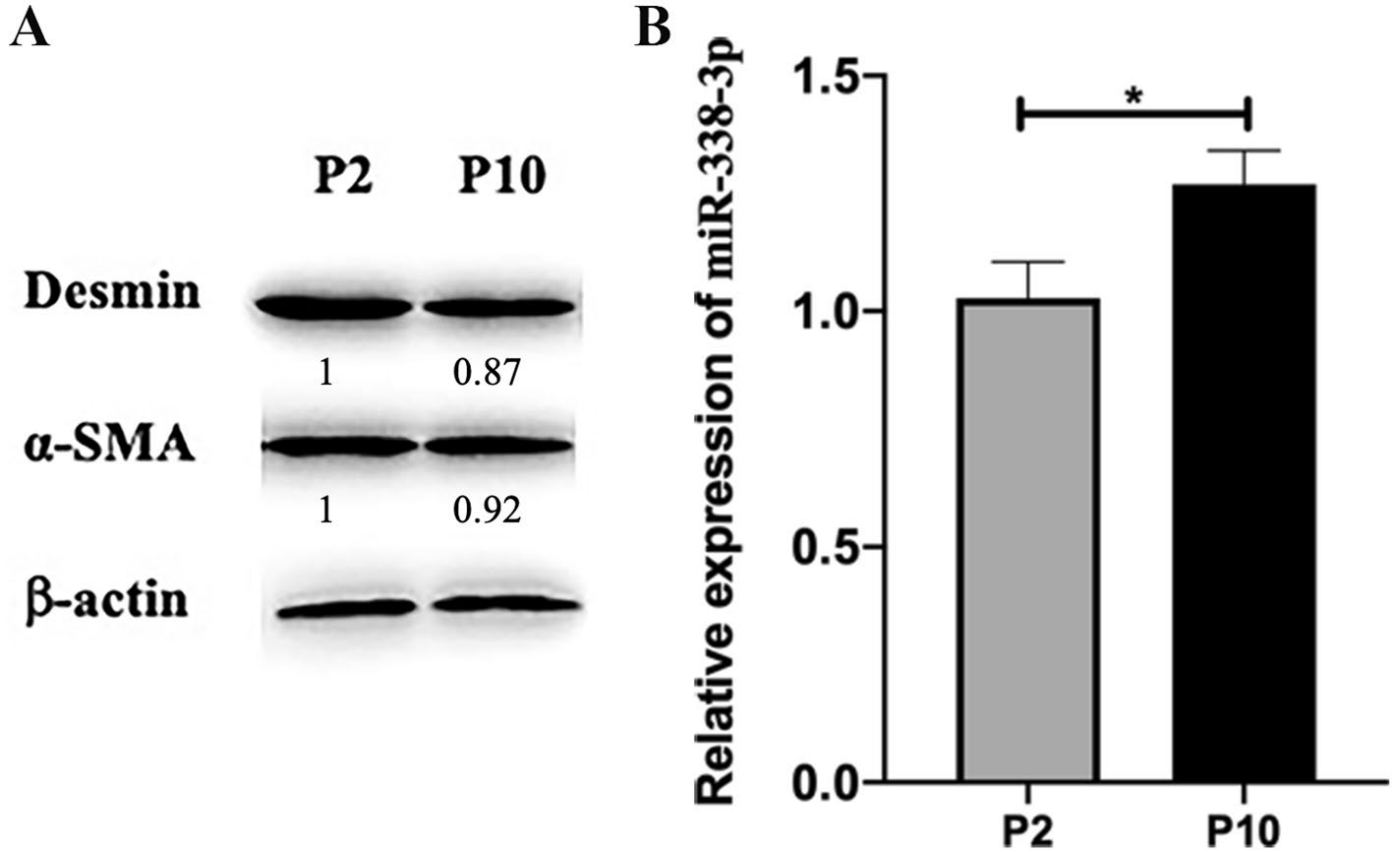
**A** Western blots analysis of selective protein markers of VSMC phenotypes using protein extractions from P2 and P10. β-actin was used as the control protein. The intensity of the bands was measured by ImageJ. Fold change relative to P2 was calculated using Desmin: β-actin ratio and α-SMA: β-actin ratio. Fold change was indicated below the corresponding band. **B** Primary VSMCs at P10 expressed a higher level of miR-338-3p than those at P2. ^*^P < 0.05

The VSMC passage number was correlated with their respective phenotypes. Specifically, at P0–P4, VSMCs were mainly contractile, while at P6 and later stages, the cells exhibited a synthetic phenotype [7, 23]. We extracted and cultured primary rat VSMCs, and then tested whether VSMCs at a later stage of passage showed a relatively higher level of miR-338-3p. VSMCs at P2 and P10 were chosen to perform Western blot and q-PCR. Selective markers were used to identify their phenotypes. As shown in Fig. 2, when compared with VSMCs at P2, cells at P10 expressed slightly lower levels of desmin and α-SMA, but a higher level of miR-338-3p (the relative expression of miR-338-3p was 1.27 ± 0.07 versus 1.03 ± 0.08).

### miR-338-3p Promotes the Phenotypic Shift of VSMCs

**Fig. 3:**
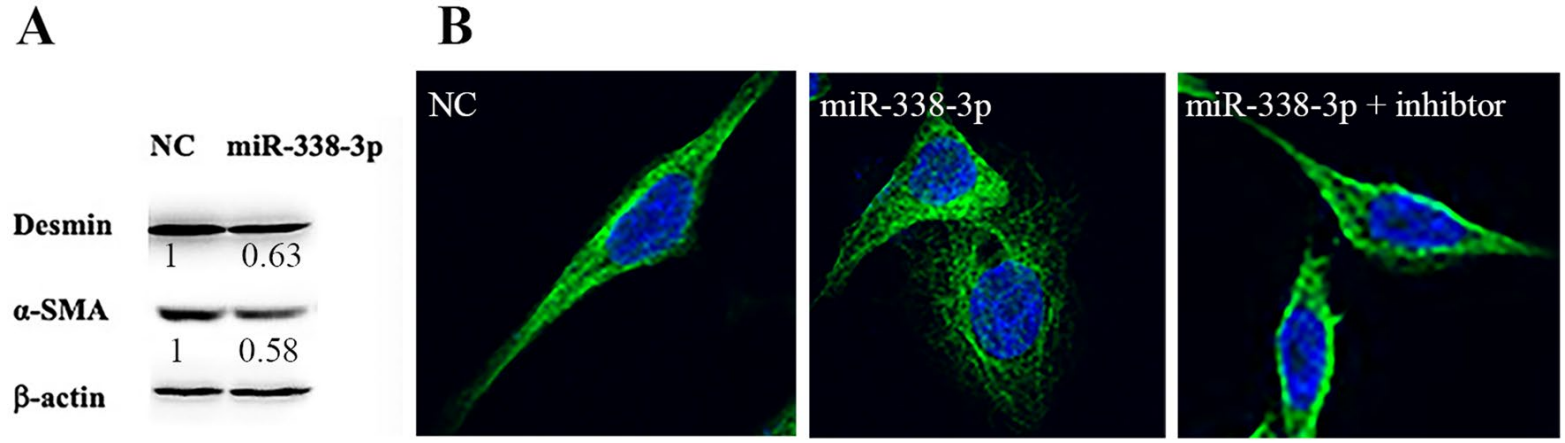
**A** VSMCs transfected with miR-338-3p mimics expressed lower levels of desmin and α-SMA. β-actin was used as the control protein. The intensity of the bands was measured by ImageJ. Fold change relative to negative control(NC) was calculated using Desmin: β-actin ratio and α-SMA: β-actin ratio. Fold change was indicated below the corresponding band. **B** Immunofluorescent staining showed that VSMCs presented a spindle-like shape. After transfection with miR-338-3p mimics, the cells became shorter in length, while co-transfection with miR-338-3p mimics and inhibitor had little influence on cell appearance

Based on the aforementioned findings, we proceeded to ask which roles miR-338-3p might play in the phenotypic shift of VSMCs. VSMCs of synthetic phenotype were characterized by their decreased expression of α-SMA and desmin. We transfected human VSMCs with equal amounts of miR-338-3p mimics or negative controls (NC). It was revealed in Fig. 3A that the miR-338-3p mimics downregulated the level of α-SMA and desmin. Immunofluorescent staining of desmin showed that VSMCs lost their spindle-like outline, and manifested with more of a fibroblast appearance when transfected with miR-338-3p mimics; while when VSMCs were co-transfected with miR-338-3p mimics and inhibitor, their spindle-like appearance was scarcely influenced (3B). These results implied that VSMCs transfected with miR-338-3p mimics were shifting towards a synthetic phenotype.

### Desmin is a Target of miR-338-3p

**Fig. 4:**
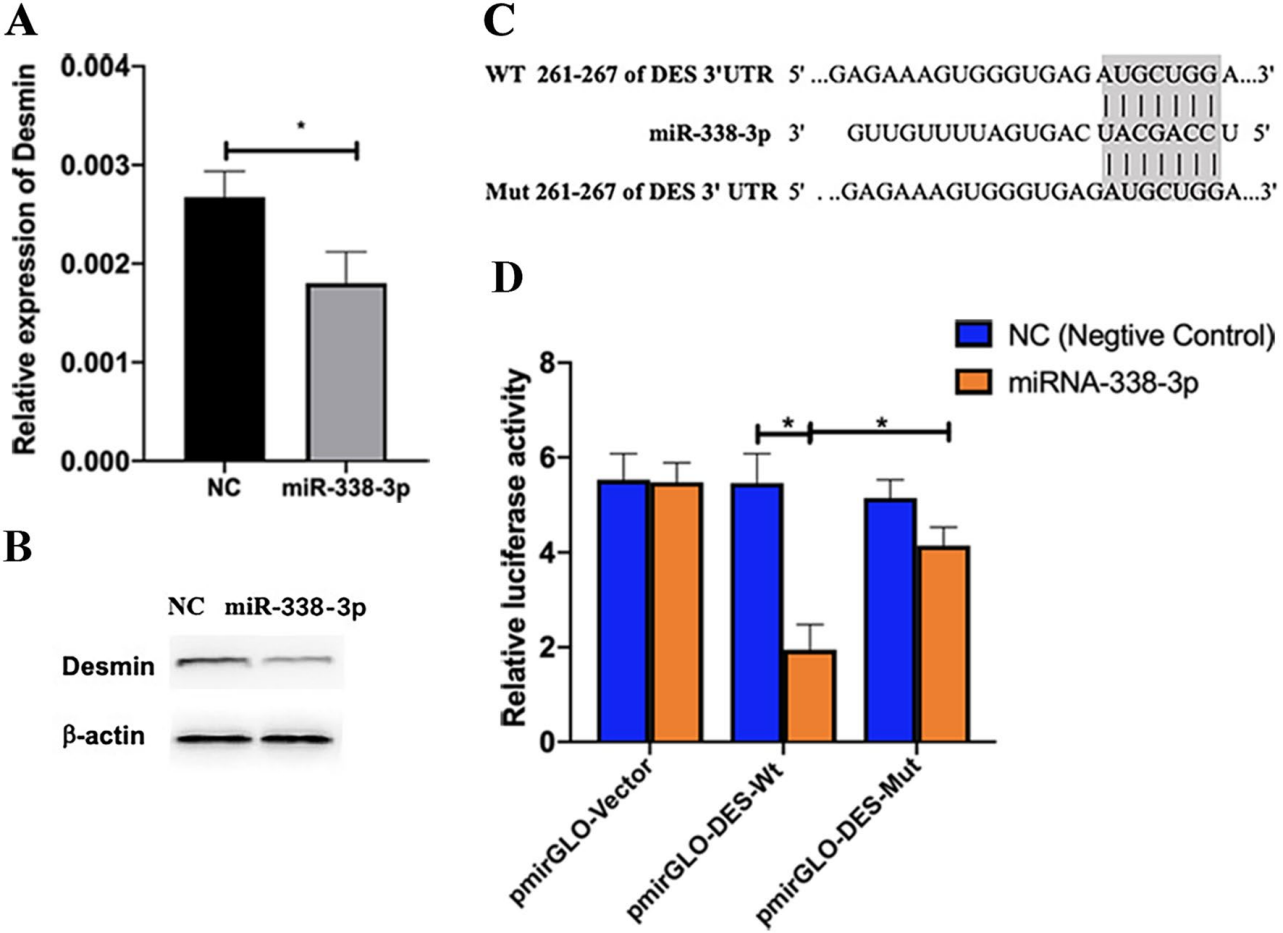
**A** and **B** miR-338-3p inhibited the expression of desmin. Desmin expression profiles were assessed by Western blot and q-PCR. ^*^P < 0.05. **C** miR-338-3p and its putative binding sequences in the 3’UTR of DES. The wild-type and mutant 3’UTR segment of DES are shown. **D** Luciferase reporter assay confirmed the direct interaction between miR-338-3p and the 3’UTR of DES

Since the loss of desmin in VSMCs is a canonical marker of a synthetic phenotype, and since bioinformatic prediction recognized desmin as one potential target of miR-338-3p, we first transfected miR-338-3p mimics in cultured human VSMCs and observed a reduction of desmin both in protein and mRNA levels by Western blot and q-PCR (Fig. 4A and B). The relative expression of DES was 0.0018 ± 0.00031 (miR-338-3p) versus 0.0027 ± 0.00026(NC), and the protein level of desmin was also significantly reduced, implying an inhibitive effect of miR-338-3p on desmin. Next, we proceeded to verify this prediction in vivo by means of a dual luciferase reporter assay system. As shown in Fig. 4C and D, the activity of luciferase was mostly inhibited only when miR-338-3p was co-transfected with wild-type desmin in 293-T cell lines, suggesting a direct interaction of miR-338-3p and the 3’-UTR of desmin.

### miR-338-3p Promotes the Proliferation But Not Migration of VSMCs

**Fig. 5:**
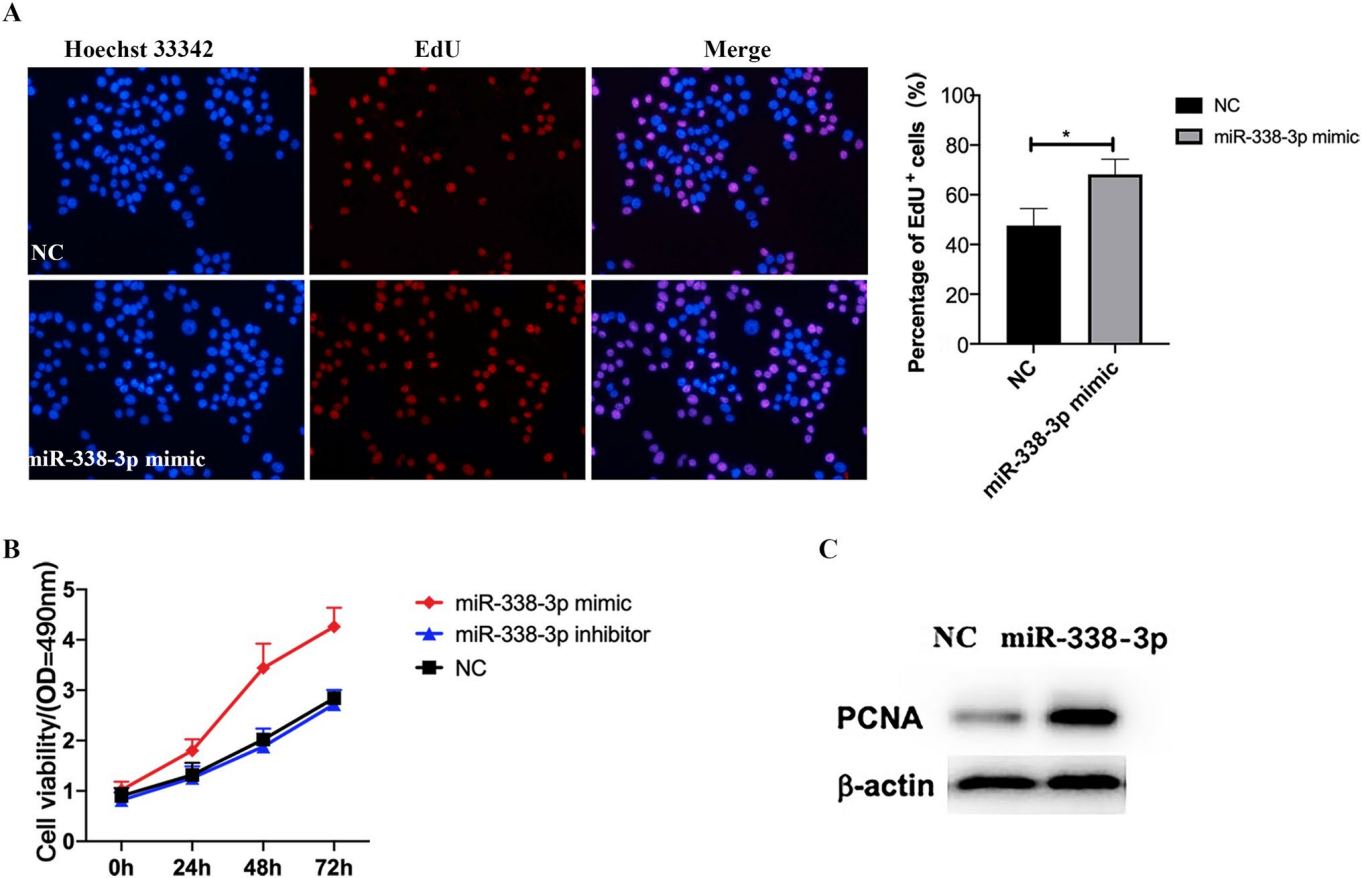
**A** Effect of miR-338-3p overexpression on the DNA synthesis of VSMCs was determined with an EdU incorporation assay. Bar graph refers to the percentage of EdU-positive cells. Data are expressed as mean ± SD (*n* = 3; ^*^P < 0.05). **B** VSMC viability was assessed via MTT assay. The optical density (OD) values were measured at 490 nm, and the OD of each group at each time point was recorded to plot the curve. **C** Proliferation-related markers were detected by Western blot

To further investigate the role of miR-338-3p in VSMC proliferation and migration, we transfected human VSMCs with miR-338-3p mimics, inhibitor, or NC, as mentioned earlier. To evaluate VSMC proliferation, the EdU incorporation assay, MTT assay, and proliferating cell nuclear antigen (PCNA) expression by Western blot were performed. As revealed in Fig. 5A, compared with NC, the miR-338-3p mimics markedly increased EdU-positive VSMCs (68.2% ± 6.1% versus 47.6% ± 6.9%), implying a promotive effect of miR-338-3p on DNA synthesis. A consistent result was obtained with the MTT assay (Fig. 5B). Compared with NC, the miR-338-3p mimics significantly increased cell viability in a time-dependent manner (the optical density [OD] value after 24 h, 48 h, and 72 h was 1.80 ± 0.22 versus 1.32 ± 0.24; 3.44 ± 0.48 versus 2.02 ± 0.13; and 4.26 ± 0.38 versus 2.84 ± 0.11, respectively), but we did not observe an obvious inhibitory effect of the miR-338-3p inhibitor (the OD value after 24, 48, and 72 h was 1.26 ± 0.23 versus 1.32 ± 0.24; 1.88 ± 0.36 versus 2.02 ± 0.13; and 2.72 ± 0.88 versus 2.84 ± 0.11, respectively). Western blot analysis of PCNA also provided supportive results (Fig. 5C). There were no changes in the mobile ability, as determined by the negative results of the wound-healing assay (Supplementary Fig. 1).

## Discussion

Atherosclerosis (AS) is a dynamic and multi-stage process. Vascular smooth muscle cells (VSMCs) play important roles in this process. Changes in VSMC structure and function form the pathological basis for AS. As the human body develops, VSMCs primarily exist in two different phenotypes: contractile and synthetic, respectively [6]. Synthetic VSMCs predominate in the embryonic and neonatal periods, and contractile VSMCs (or differentiated cell types) are mainly found in the vessels of adults [7–10]. Cytoskeletal changes in contractile VSMCs include an increase in the α isoform of actin and the appearance of desmin [24, 25]. However, under some pathological conditions, these cells are able to return to a synthetic phenotype (or a dedifferentiated state), which is usually an early event in atherogenesis.

The mechanism by which VSMCs established the phenotypic shift in AS has yet to be determined. Since miR-338-3p has long been reported to be involved in cardiovascular physiopathology, we asked if miR-338-3p participated in the process of AS [20–22]. In this study, we established an AS rat model, and confirmed that miR-338-3p was upregulated in AS using tissues obtained from ApoE^−/−^ rats. Further, we extracted and cultured primary rat VSMCs. VSMCs at a later stage of passage, representative of a synthetic phenotype, showed a relatively higher level of miR-338-3p. So far, we determined that in AS and synthetic VSMCs, miR-338-3p was upregulated.

Then, we proceeded to explore the possible roles that miR-338-3p might play in the process of AS. A series of experiments were designed to determine how are phenotype, as well as proliferative and mobile abilities affected by miR-338-3p. As for the detection of phenotype, we employed widely accepted cytoskeletal markers, including desmin and α-SMA. Western blotting of proteins from rat aorta tissues showed no difference between wild-type and ApoE^−/−^ rats. Considering that aorta tissues contain other types of cells except for VSMCs, human VSMCs were cultured and transfected with miR-338-3p mimics for further analysis. Western blot showed that the miR-338-3p mimics markedly downregulated the level of these two proteins, suggestive of a switch into a synthetic phenotype. Immunofluorescent staining provided a morphological comparison, where it was identified that miR-338-3p mimics alleviated the spindle-like or sharp appearance. When miR-338-3p inhibitor recovered the appearance, it means this change was miR-338-3p-specific.

We speculated that these observations were associated with the inhibitory effect of miRNAs on targeted genes, and *DES* (encoding desmin) and/or *ACTA2*(α-SMA) might be these target genes. Then we used the TargetScan (http://www.targetscan.org/) and miRDB (http://mirdb.org) databases to predict the potential targets of miR-338-3p. We found that both tools suggested *DES* was a target of miR-338-3p. Desmin is a muscle-specific intermediate filament protein and has been implicated in smooth muscle contraction and cell migration [26–28]. Using a dual luciferase reporter assay system, we confirmed that desmin was a direct target of miR-338-3p.

In the detection of VSMCs proliferation, multiple methods were employed. miR-338-3p is a widely studied small RNA in cancer, and consistently, it is down-regulated and plays tumor suppressor roles in multiple cancers, such as neuroblastoma, gastric cancer, colorectal carcinoma, lung cancer and hepatocellular carcinoma, where cancer cell proliferation is inhibited by miR-338-3p [29, 30]. Unexpectedly, we found that miR-338-3p exerted a positive effect on VSMCs proliferation, which was confirmed by multiple methods including EdU incorporation assay and MTT assay. This is in contrast with the current studies in cancer. We suppose this result may be due to the fact that cancer cells are different from normal cells in nature. When a cell become cancerous, dozens of mutations accumulate in the various genes that control cell proliferation, resulting in malignant proliferation; while VSMCs proliferation, to a certain extent, is a compensatory and protective reaction to injury in AS [31]. On the other hand, the promotive effect on VSMCs proliferation may have been independent of its inhibitory effect on the desmin gene. A recent study on AS verified that miR‑338‑3p directly targeted TET2 and negatively regulated the expression of TET2 [32]. TET2, Tet methylcytosine dioxygenase 2, exerts function via catalyzing the oxidation of DNA 5-methylcytosine and is involved in cell apoptosis [33]. Previous studies showed that TET2 was downregulated during the pathogenesis of atherosclerosis [34]. The information might provide clues to this question and we will continue to explore in subsequent research.

Based on the fact that miR-338-3p inhibited the expression of desmin, we speculated that the mobile abilities of VSMCs might be weakened by miR-338-3p. We adopted a simple method, such as the wound healing assay, to analyze VSMC mobility. Multiple assays featuring 24-h transfection repeatedly brought about negative results. Considering that this treatment time might not be enough to induce a significant change, we prolonged it to 48 h. However, this still did not yield a different observation. We concluded that this effect was associated with the decreased expression of desmin.

In conclusion, we found in this study that miR-338-3p was a driving factor for the development of AS. In terms of its associated mechanism, miR-338-3p repressed the expression of desmin, which is an important element for the structural integrity and function of VSMCs. The loss of desmin then partly accounts for the phenotype change into a “synthetic” phenotype. Simultaneously, increased miR-338-3p promoted the proliferation of VSMCs independent of its inhibition on desmin. So, there may be other mechanisms underlying how miR-338-3p exhibited promotive effects on cell proliferation. miR-338-3p didn’t affect the mobile abilities of VSMCs.

## Supplementary Information

Below is the link to the electronic supplementary material.

Supplementary file1 — Supplementary Figure 1Wound Healing assay. VSMCs were plated in six-well plates at a density of 4×105 cells/well. After adherence, cells were transfected with the indicated expression vectors. The monolayer was wounded with a plastic tip and monitored under bright-field microscope. VSMCs migration to the wounded gap was then monitored by microscopy after 24 hours and 48 hours. The images are representative of three independent experiments that gave similar results. The results were expressed as a percentage of wound closure. ns: no significance. (TIF 1314 kb)

Supplementary file2 (DOCX 52 kb)
