# Supplementary figures and images for: Micro-RNA-338-3p Promotes the Development of Atherosclerosis by Targeting Desmin and Promoting Proliferation
Source: Mol Biotechnol. 2021 Jun 7;63(9):840–8. doi: 10.1007/s12033-021-00341-8 (PMC8316222; doi:10.1007/s12033-021-00341-8)

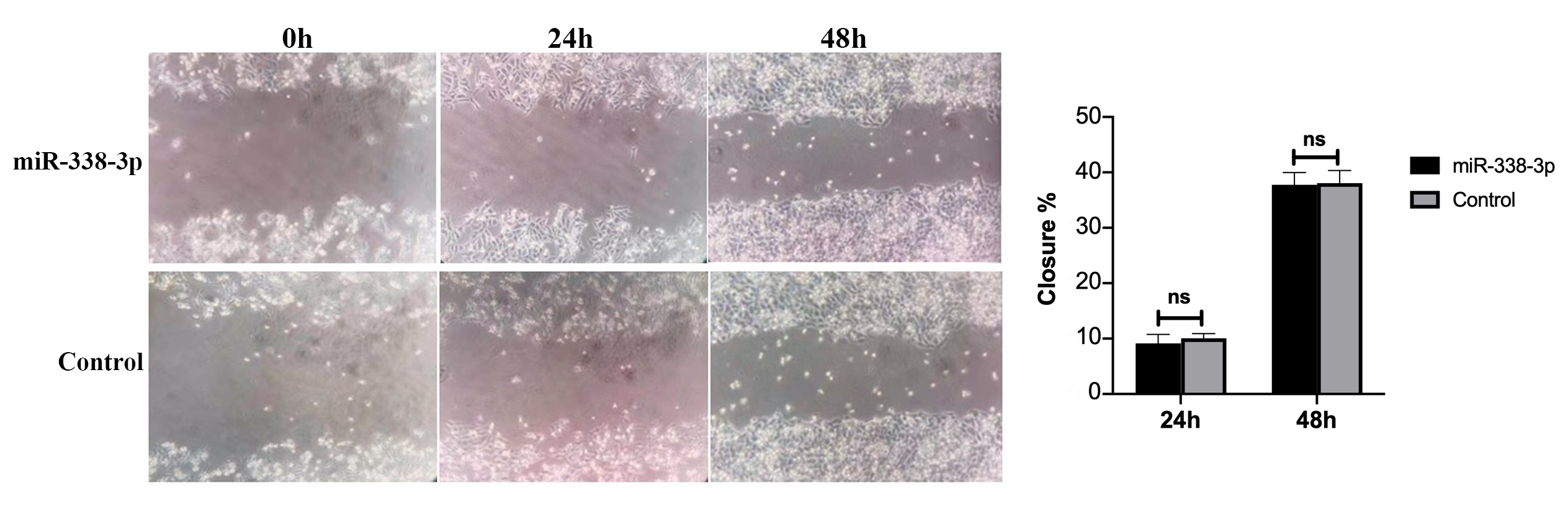

Supplement: Supplementary file 1 — Supplementary file1 — Supplementary Figure 1Wound Healing assay. VSMCs were plated in six-well plates at a density of 4×10^5^ cells/well. After adherence, cells were transfected with the indicated expression vectors. The monolayer was wounded with a plastic tip and monitored under bright-field microscope. VSMCs migration to the wounded gap was then monitored by microscopy after 24 hours and 48 hours. The images are representative of three independent experiments that gave similar results. The results were expressed as a percentage of wound closure. ns: no significance. (TIF 1314 kb) [file 12033_2021_341_MOESM1_ESM.tif]
