# Supplementary material for: Micro-RNA-338-3p Promotes the Development of Atherosclerosis by Targeting Desmin and Promoting Proliferation
Source: Mol Biotechnol. 2021 Jun 7;63(9):840–8. doi: 10.1007/s12033-021-00341-8 (PMC8316222; doi:10.1007/s12033-021-00341-8)
Supplement: Supplementary file 2 — Supplementary file2 (DOCX 52 kb) [file 12033_2021_341_MOESM2_ESM.docx]

Supplementary

Primers for q-PCR

| Desmin | Forward | 5’-TCGGCTCTAAGGGCTCCTC-3’ |
| --- | --- | --- |
|  | Reward | 5’-CGTGGTCAGAAACTCCTGGTT-3’ |
| GAPDH | Forward | 5’-TGTGGGCATCAATGGATTTGG-3’ |
|  | Reward | 5’-ACACCATGTATTCCGGGTCAAT-3’ |
| miR-338- 3 | Forward | 5’-GGGGTACCGAATCTTCCCAGTAGGCG-3’ |
|  | Reward | 5’-TTGCGGCCGCAAAGGAGAAGGGCCAAAC-3’ |
| U6 | Forward | 5’- CGCTTCGGCAGCACATATACTAA-3’ |
|  | Reward | 5’- TATGGAACGCTTCACGA ATTTGC-3’ |
